# An In Vitro Study on the Combination Effect of Metformin and N-Acetyl Cysteine against Hyperglycaemia-Induced Cardiac Damage

**DOI:** 10.3390/nu11122850

**Published:** 2019-11-21

**Authors:** Rabia Johnson, Nonhlakanipho F. Sangweni, Sihle E. Mabhida, Phiwayinkosi V. Dludla, Lawrence Mabasa, Sylvia Riedel, Charna Chapman, Rebamang A. Mosa, Abidemi P. Kappo, Johan Louw, Christo J. F. Muller

**Affiliations:** 1 Biomedical Research and Innovation Platform (BRIP), South African Medical Research Council, Tygerberg 7505, South Africasihlemabhida@gmail.com (S.E.M.); Lawrence.Mabasa@mrc.ac.za (L.M.); Sylvia.Riedel@mrc.ac.za (S.R.); Charna.Chapman@mrc.ac.za (C.C.); Johan.Louw@mrc.ac.za (J.L.);; 2 Division of Medical Physiology, Faculty of Health Sciences, Stellenbosch University, Tygerberg, Cape Town 7505, South Africa; 3 Department of Biotechnology, University of the Western Cape, Cape Town 7505, South Africa; 4 Department of Biochemistry, Genetics and Microbiology (BGM), Division of Biochemistry, University of Pretoria, Hatfield 0028, South Africa; 5 Department of Biochemistry and Microbiology, University of Zululand, Kwadlangezwa, Durban 3800, South Africa

**Keywords:** oxidative stress, diabetes, apoptosis, N-acetyl cysteine, metformin, cardiovascular

## Abstract

Chronic hyperglycaemia is a major risk factor for diabetes-induced cardiovascular dysfunction. In a hyperglycaemic state, excess production of reactive oxygen species (ROS), coupled with decreased levels of glutathione, contribute to increased lipid peroxidation and subsequent myocardial apoptosis. N-acetylcysteine (NAC) is a thiol-containing antioxidant known to protect against hyperglycaemic-induced oxidative stress by promoting the production of glutathione. While the role of NAC against oxidative stress-related cardiac dysfunction has been documented, to date data is lacking on its beneficial effect when used with glucose lowering therapies, such as metformin (MET). Thus, the aim of the study was to better understand the cardioprotective effect of NAC plus MET against hyperglycaemia-induced cardiac damage in an H9c2 cardiomyoblast model. H9c2 cardiomyoblasts were exposed to chronic high glucose concentrations for 24 h. Thereafter, cells were treated with MET, NAC or a combination of MET and NAC for an additional 24 h. The combination treatment mitigated high glucose-induced oxidative stress by improving metabolic activity i.e. ATP activity, glucose uptake (GU) and reducing lipid accumulation. The combination treatment was as effective as MET in diminishing oxidative stress, lipid peroxidation and apoptosis. We observed that the combination treatment prevented hyperglycaemic-induced cardiac damage by increasing GLUT4 expression and mitigating lipid accumulation via phosphorylation of both AMPK and AKT, while decreasing nuclear factor kappa-light-chain-enhancer of activated B cells (NF-kB), as well as protein kinase C (PKC), a known activator of insulin receptor substrate-1 (IRS-1), via phosphorylation at Ser307. On this basis, the current results support the notion that the combination of NAC and MET can shield the diabetic heart against impaired glucose utilization and therefore its long-term protective effect warrants further investigation.

## 1. Introduction

According to the latest IDF report (2017), the global prevalence of diabetes is rising all over the world [1]. This rising diabetes epidemic contributes significantly to the aetiology of cardiovascular dysfunction, with diabetic individuals presenting increasingly with heart failure (HF) [2]. A large body of evidence suggests that chronic hyperglycaemia, linked to augmented reactive oxygen species (ROS) levels and oxidative stress, plays a major role in the development and progression of deteriorated cardiac function [3,4,5,6]. For example, in the diabetic heart, aberrant glucose auto-oxidation and shifts in redox balance are known to decrease tissue concentration of glutathione (GSH) and impair antioxidant defence enzymes, such as superoxide dismutase (SOD) and catalase (CAT) [5,7]. Consequently, large amounts of mitochondrial ROS are produced that promote the degeneration of biological macromolecules, propagating lipid peroxidation while decreasing metabolic activity. Furthermore, in the failing heart free fatty acids become the preferred energy substrate, resulting in increased formation of diacylglycerol (DAG) [5,8]. Diacylglycerol, in turn, activates nuclear factor-kappa B (NF-kB) and protein kinase C (PKC) which later blunts peripheral glucose uptake (GU) by phosphorylating insulin receptor substrate 1 (IRS-1) at Ser307 and prevents insulin-mediated phosphorylation of protein kinase B (AKT)Thr473, while diminishing glucose transporter (GLUT) 4 uptake from peripheral tissue [9]. Altogether, cytosolic adenosine triphosphate (ATP) production is diminished, which furthermore exacerbates mitochondrial depolarization and increases the release of cytochrome C, caspase 3/7 activity and subsequent cardiac apoptosis. At present, commonly used antidiabetic drugs such as metformin are known to present limited efficacy in protecting against the aforementioned diabetes associated cardiac complications.

Metformin is an insulin sensitizing agent known to partially protect the heart from high glucose induced oxidative stress [10]. However, as a result of the continuous increase in deaths of diabetic patients, it is presumed the long-term effect of MET to protect the diabetic myocardium diminishes over time. For this reason, supplementation of glucose lowering drugs with natural antioxidants is increasingly being explored to improve cardiac health. Various studies have focused on the use of N-acetylcysteine (NAC) to reduce oxidative stress, as recently reviewed [11]. In the later review, scientific evidence is provided confirming that NAC is a potent antioxidant and, as a precursor of GSH, has attracted great interest as a ROS scavenger [11,12]. NAC has been shown to improve atrial fibrillation (AF), decrease ischaemic reperfusion injury and potentiate the effect of angiotensin-converting enzymes in myocardial infarction episodes [13,14]. Furthermore, treatment with NAC is known to improve cardiac remodelling by decreasing ROS-mediated NF-kB-activation, which contributes to decreased GU, mitochondrial dysfunction and subsequent apoptosis of the stressed myocardium [15]. However, to date, limited studies are available on the ability of NAC to decrease intracellular oxidative stress when used in combination with the glucose lowering drug MET. Thus, this study aims to investigate the protective effect for the combined use of NAC and MET, as well as the mechanism employed to improve GU in an in vitro H9c2 cardiomyoblast model.

## 2. Materials and Methods

### 2.1. Metformin and N-Acetylcysteine Preparation

A 10 mM stock solution of both MET and NAC (both supplied by Sigma-Aldrich, Saint Louis, MO, USA) was made in tissue culture (TC) grade water (Lonza, Walkersville, MD, USA) and thereafter, 2 mM working stock solutions were prepared and filter-sterilised. Final concentrations of 1 µM MET and/or 1 mM NAC were prepared in Dulbecco’s Modified Eagle Medium (DMEM) (supplemented with 1% *v*/*v* foetal bovine serum (FBS)) and containing 33 mM glucose (Lonza BioWhittaker, Verviers, Belgium) for subsequent experimental analysis.

### 2.2. Effect of MET and NAC on H9c2 Cells Exposed to High Glucose

Rat heart derived ventricular H9c2 (ATCC, CRL-1446) cardiomyoblasts, obtained from the American Type Culture Collection, are immortalized cells with a cardiac phenotype. The H9c2 cells are extensively used to study cardiovascular dysfunction caused by prolonged high glucose exposure [16,17]. Briefly, H9c2 cells were cultured in DMEM (supplemented with 10% *v*/*v* FBS) under standard tissue culture conditions (37 °C in humidified air and 5% CO_2_) in a 75 cm^2^ flask and media was refreshed every two days. Upon 80% confluency, cells were split and seeded in DMEM for 48 h in either a 6-well plate (2 × 10^5^ cells/well) for protein, 96-well plate (0.8 × 10^5^ cells/well) for ATP or 24-well plate (1 × 10^5^ cells/well) for all other analyses. Subsequently, H9c2 cells were cultured in glucose-free DMEM without phenol red (Sigma-Aldrich, Saint Louis, MO, USA), supplemented with 1% bovine serum albumin (BSA) for 30 min, before hyperglycaemia was induced by exposing H9c2 cardiomyoblasts to 33 mM glucose (high glucose; HG) for 24 h, as per the method described by Jadaun et al. [16]. The following day, to assess the ability of NAC plus MET to attenuate high glucose-induced cardiac injury, H9c2 cardiomyoblasts were treated with either 1 mM NAC, 1 µM MET or a combination of NAC plus MET for 24 h. Cells exposed to either normal glucose (NG; 5.5 mM) or HG were treated with the vehicle control. All treatment doses were based on results obtained from previous studies [4,7,18,19,20,21].

### 2.3. Measurement of Metabolic Activity

The ViaLight^™^ plus Adenosine Triphosphate (ATP) kit (Lonza, Basel, Switzerland), was used as a rapid screening method to measure metabolic activity and cytotoxicity, as per manufacturer’s instructions. Briefly, cells cultured in white 96-well plates were removed from the incubator after the predetermined treatment conditions. Next, 50 µL of the culture media remained and 50 µL of the cell lysis buffer, provided in the kit, was added to each well and incubated for 10 min at room temperature. Thereafter, 100 µL of AMR plus solution was added to each well and incubated for an additional 2 min. Luminescence was quantified using the BioTek FL×800 plate reader and analysed with the Gen 5 software (Bio-Tek Instruments Inc., Winooski, VT, USA).

### 2.4. Measurement of 2-Deoxy-[3H]-D-Glucose (DOG) Uptake

Radiolabelled 2-Deoxy-[3H]-D-glucose (DOG) uptake was measured in H9c2 cells to assess myocardial glucose uptake. The principle behind the assay entails the addition of radioactively labelled DOG to H9c2 cardiomyoblasts, followed by quantifying DOG by means of a scintillation counter. To assess NAC and MET ability to improve GU in H9c2 cardiomyoblasts after high glucose exposure, DOG uptake was performed as per the previously published protocol [7]. Briefly, after the predetermined treatment, H9c2 cells were exposed to 0.5 µCi/mL 3H-2-DOG and 2% BSA for 15 min in a 24-well tissue culture plates. Thereafter, cells were lysed with 0.1 M NaOH/1% SDS and incubated for an additional 45 min at 37 °C. An aliquot was used for protein determination and the remaining cell lysates were then added to scintillation vials containing 1 mL tissue culture (TC) grade water. Subsequently, 8 mL of Ready Gel Ultima Gold was pipetted into scintillation vials and equilibrated overnight at room temperature, where after DOG was assessed in a liquid scintillation analyser (2200 CA, Parkard Tricarb series) by liquid scintillation (PerkinElmer, Downers, Crove, IL, USA). Results obtained were then calculated as previously described and expressed in arbitrary units [7].

### 2.5. Quantification of Intracellular Lipid Content

Cardiac lipid accumulation is associated with decreased cardiac function. To quantify lipid accumulation, Oil red O (ORO) staining (Sigma-Aldrich, St Louis, MO, USA), a widely used lysochrome (fat-soluble) diazo dye for staining and quantifying neutral intracellular lipids, was adapted as previously described [22]. Briefly, following the relevant treatment conditions, H9c2 cells were fixed with 4% (*v*/*v*) paraformaldehyde for 15 min and washed three times in PBS. Thereafter, cells were incubated in freshly prepared 0.7% (*v*/*v*) ORO staining solution for 30 min at room temperature. Following a 5 min rinse in distilled water, cells were immersed in 100% isopropanol for 20–30 s to extract the dye. Supernatants were then transferred to a 96-well plate for measurement of optical density for lipid accumulation at 490 nm with a BioTek EL×800 plate reader using Gen 5 software. For normalisation, cells were treated with 70% ethanol, then the solution was aspirated before the addition of 400 µL crystal violet (CV) for 5 min at room temperature. The CV was removed, and cells washed with PBS. Thereafter, 70% ethanol was added to extract the CV and 100 µL transferred to a clean 96-well plate for the measurement of optical density at 570 nm.

### 2.6. Measurement of Intracellular Reactive Oxygen Species (ROS)

Intracellular production of ROS was detected using 2’,7’-dichlorflourescein diacetate (DCFH-DA) fluorescent dye (Cell Biolabs Inc., San Diego, CA, USA), as per the previously described protocol [7]. Briefly, intracellular production of ROS was evaluated after H9c2 cells were exposed to 100 μL of 1 µM DCFH-DA. Thereafter the cells were incubated under standard TC conditions for 30 min. Next, HBSS was added and the DCFH-DA fluorescent intensity was measured at an excitation and emission wavelength of 485 ± 20 nm/ 528 ± 20 nm by means of a BioTek FL×800 plate reader and analysed using Gen 5^®^ software.

### 2.7. Measurements of Superoxide Dismutase (SOD) Activity

Superoxide dismutase (SOD) is an endogenous antioxidant which scavenges the superoxide anion into hydrogen peroxide and oxygen. SOD activity was measured by means of a Biovision SOD activity kit (Mountain View, CA, USA), according to manufacturer’s instructions. Briefly, H9c2 cardiomyoblasts were lysed by the addition of 100 µL of 0.1 M trizma/hydrochloride (Tris/HCl) and 0.1 mg/mL phenylmethylsulfonyl fluoride (PMSF). Lysed cells were then centrifuged at 15,000× *g* for 5 min at 4 °C. Twenty microliter of the cell lysates were then transferred to a fresh 96-well plate with the addition of 200 µL WST and 20 µL Enzyme Working Solution (supplied in the kit), where after the cells were incubated at 37 °C for 20 min prior to measuring of SOD activity. Absorbance was read at an excitation and emission wavelength of 485 ± 20 nm/528 ± 20 nm with a Biotek ELX 800 plate reader using Gen 5 software.

### 2.8. Measurements of Glutathione (GSH) Content

The Glutathione Luminescent-based assay is an assay design for the quantification and detection of glutathione (GSH) levels, a known antioxidant. Total glutathione content was assessed using a 7-amino-4-chloromethylcoumarin CellTrackerTM Blue dye (Invitrogen Corporation, Carlsbad, CA, USA) assay kit following the manufacturer’s guidelines. Briefly, cells cultured in white 96-well TC plates, were lysed in 50 µL GSH lysis reagent. Cells were mixed using a orbital shaker for 5 min, where after 50 µL of a luciferin generation reagent was added and cells incubated for an additional 15 min at room temperature. GSH fluorescent intensity was determined using the BioTek FL×800 plate reader with an excitation at 485 ± 20 nm and emission at 528 ± 20 nm.

### 2.9. Lipid Peroxidation Analysis

The thiobarbituric acid reactive substances (TBARS) assay is an established method that quantifies lipid peroxides based on the production of a reactive aldehyde, malondialdehyde (MDA). Lipid peroxidation was assessed by using an OxiSelect^™^ TBARS assay kit, as previously described [7,23]. Briefly, H9c2 cells were scraped using a rubber policeman and resuspended in 1 mL DPBS containing 10 µL of butylated hydroxytoluene, before being homogenized for 1 min. Thereafter, 100 µL of sodium dodecyl sulfate lysis solution was pipetted into new eppendorfs containing 100 µL of standards and the treated samples (H9c2 cell lysate). The sample mixtures were vortexed and incubated for 5 min at room temperature. Subsequently, 250 μL of thiobarbituric acid reagent was pipetted into each tube, thoroughly mixed and incubated for 45–60 min at 95 °C before centrifuging for 15 min at 1500× *g*. Next, supernatants of samples and standards were inserted into a new 96-well plate to measure the absorbance at 532 nm using a BioTek ELX800 plate reader (Gen 5 software).

### 2.10. Evaluating Mitochondrial Membrane Potential (ΔΨm)

JC-1 stain was used to assess mitochondrial membrane depolarization, as previously described [7,23]. Briefly, treated H9c2 cardiomyocytes were washed with warm DPBS before staining with 2 μM JC-1 solution in DMEM without phenol red, and then incubated at standard tissue culture conditions for 30 min in the dark. Thereafter, cardiomyocytes were washed with warm DPBS and the fluorescence was measured at Excitation of 485 ± 20 nm and dual Emission of 530 ± 25 nm and 590 ± 35 nm, using a BioTek FL×800 plate reader (BioTek Instruments Inc., Winooski, VT, USA) and fluorescence quantified using Gen 5 software.

### 2.11. The Activity of Caspase 3/7

Caspase 3/7 activity was determined as per previously described protocol [7]. Briefly, treated cells were lysed and 20 µL of cell lysates were transferred to a 96-well plate and mixed with 20 µL of Caspase-Glo reagent (Promega, Madison, WI, USA) before the reaction was incubated for 30 min in the dark. A BioTek FLX 800 plate reader with Gen 5 software was used to measure luminescence. Data were normalised to previously acquired protein content using the reducing agent and detergent compatible (RC DC^™^) protein assay (Bio-Rad, Hercules, CA, USA).

### 2.12. Western Blot Analysis

Protein expression was determined by Western blot analysis as previously described [7]. Briefly, 40 µg of protein lysate was denatured and loaded onto a 10% SDS-polyacrylamide gel and transferred to a polyvinylidene fluoride membrane. Nonspecific binding on the membranes was blocked using 5% w/v low-fat milk in Tris-buffered saline with Tween-20. Subsequently, the membrane was incubated overnight at 4 °C with the relevant Cell Signaling (Beverly, MA, USA) primary antibodies: Phosphorylated Protein kinase B at Ser 473 (p-AKTSer473; 1:1000), phosphorylated 5’ AMP-activated protein kinase at threonine 174 (p-AMPKThr174; 1:800), phosphorylated nuclear factor-kappa β phosphorylation at serine 536 (p-NF-kBSer536, 1:1000), phosphorylated Insulin receptor substrate-1 phosphorylation at serine 307 (p-IRS-1Ser307, 1:1000), protein kinase C phosphorylation at serine 660 (p-PKCSer660, 1:1000) and GLUT4 (1:800) (Sigma-Aldrich, Saint Louis, MO, USA). The relevant horseradish peroxidase-conjugated secondary antibodies were applied the following day for 90 min at room temperature. β-Actin (1:2000) (Santa Cruz Biotechnology, Dallas, TX, USA) antibody was added as a loading control. Proteins were detected and quantified using a Chemidoc-XRS imager and Quantity One software (Bio-Rad Laboratories, Hercules, CA, USA).

## 3. Results

### 3.1. The Combination Effect of MET and NAC on ATP Activity

**Figure 1:**
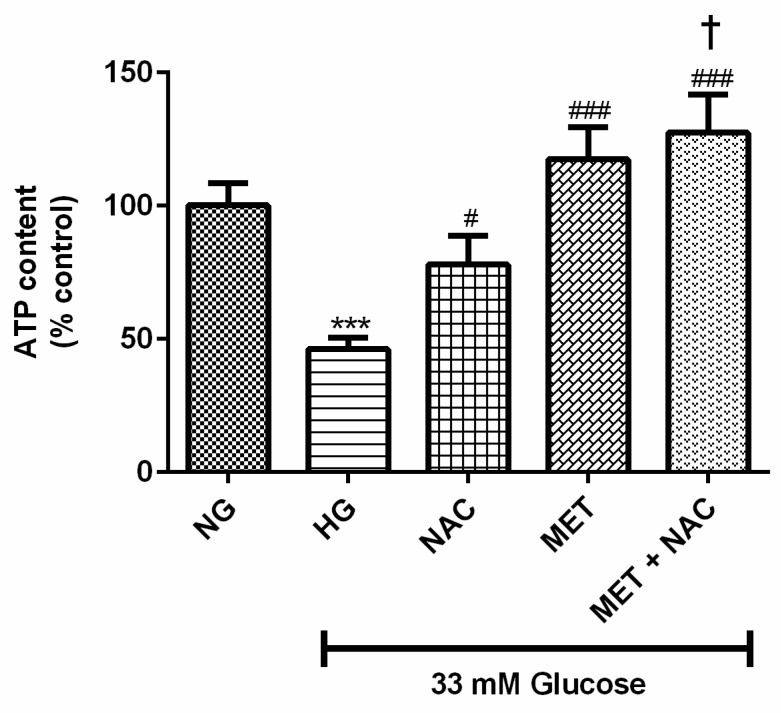
The combination effect of Metformin (MET) and N-acetylcysteine (NAC) on high glucose-induced metabolic activity. H9c2 cardiomyoblasts were cultured in high glucose (HG; 33 mM) for 24 h. Thereafter, cardiomyoblasts were treated with NAC (1 mM), metformin (MET) (1 μM) and a combination of MET+NAC. Cells treated with normal glucose (NG) served as the vehicle control. Data are presented as the mean ± SEM of 3 distinct biological experiments with each experiment having 3 technical repeats (*n* = 9). Significance is depicted as *** *p* ≤ 0.001 versus NG control, # *p* < 0.05, ### *p* ≤ 0.001 versus HG control and † *p* < 0.05 versus NAC only.

ATP, as a measurement of cellular metabolic activity, was used to assess the effect of high glucose (HG; 33 mM) on H9c2 cardiomyoblasts over a period of 24 h. Cells cultured in HG displayed a significant decrease in ATP activity compared to the normal glucose (NG; 5.5 mM) control (46% vs. 100%; *p* < 0.001). Treatment with either NAC (77% vs. 46%; *p* < 0.05), MET (118% vs. 46%; *p* < 0.001) or MET+NAC (128% vs. 46%; *p* < 0.001) was able to significantly improve the metabolic activity of cardiomyoblasts exposed to HG. The combination of MET+NAC was as effective as the MET treatment and more effective than NAC alone (128% vs. 77%; *p* < 0.05) (Figure 1).

### 3.2. The Combination Effect of MET and NAC on Glucose Uptake (GU)

**Figure 2:**
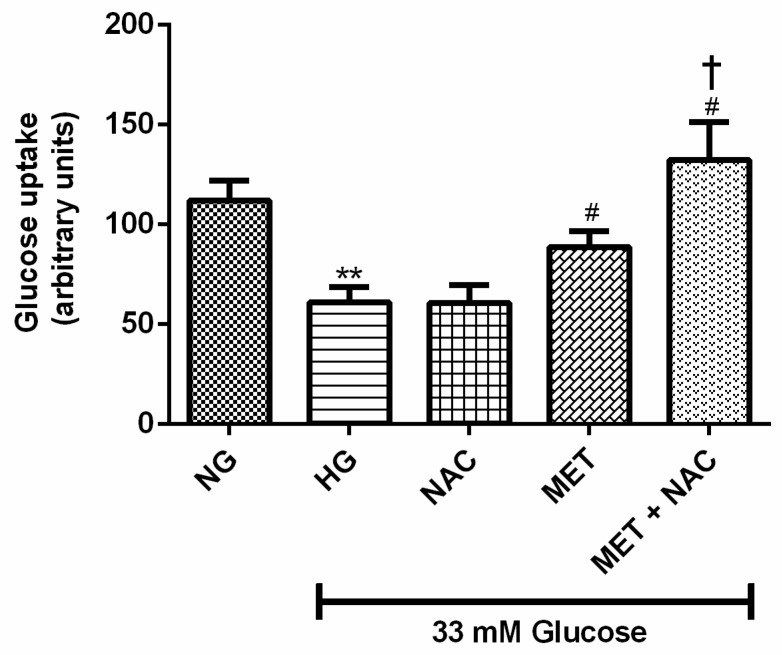
The combination effect of Metformin (MET) and N-acetylcysteine (NAC) on high glucose-induced H9c2 cells glucose uptake. H9c2 cardiomyoblasts were cultured in high glucose (HG; 33 mM) for 24 h. Cardiomyoblasts were then co-treated with NAC (1 mM), metformin (MET) (1 μM) and a combination of MET+NAC. Cells treated with normal glucose (NG) served as the vehicle control. Data are presented as the mean ± SEM of 3 distinct biological experiments with each experiment having 3 technical repeats (*n* = 9). Significance is depicted as ** *p* ≤ 0.01 versus NG control, # *p* < 0.05 versus HG control † *p* < 0.05 versus NAC only.

Impaired cardiac glucose regulation is closely linked with the development of HF. Glucose uptake was measured in H9c2 cardiomyoblasts exposed to HG levels and treated with or without NAC, MET and MET+NAC. As anticipated, GU was decreased in H9c2 cells exposed to HG when compared to the NG control (83%; *p* < 0.01). No significant difference was observed in cells exposed to NAC in comparison to the HG control. Interestingly, enhanced GU was displayed by cells treated with the combination of MET+NAC when compared to the HG control (116%, *p* < 0.05) as well as the cells treated with MET alone (46%, *p* < 0.05) (Figure 2). The combination of MET+NAC was better than NAC alone.

### 3.3. The Combination Effect of MET and NAC on Lipid Content

**Figure 3:**
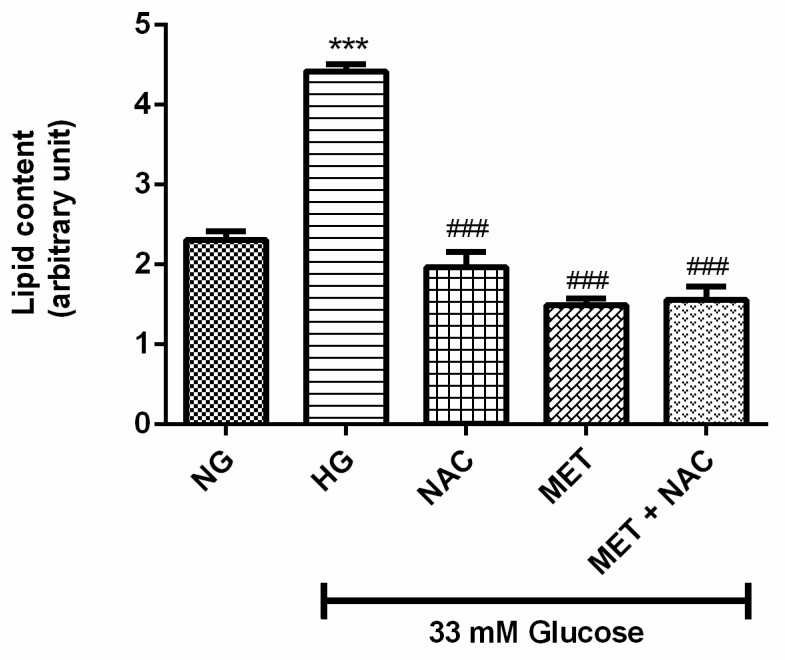
The combination effect of Metformin (MET) and N-acetylcysteine (NAC) to reverse high glucose-induced lipid accumulation. H9c2 cardiomyoblasts were cultured in high glucose (HG; 33 mM) for 24 h. Cells were then co-treated with NAC (1 mM), metformin (MET) (1 μM) and a combinatory treatment of MET+NAC. Cells treated with normal glucose (NG) served as the vehicle control. Results are expressed as the mean ± SEM of 3 independent biological experiments with each experiment having 3 technical replicates (*n* = 9). Significance is depicted as *** *p* ≤ 0.001 versus NG control, ### *p* ≤ 0.001 versus HG control.

Increase in cellular lipid accumulation can lead to cardiac dysfunction. H9c2 cardiomyoblasts exposed to HG displayed a significant increase in lipid content in comparison to the NG control (91% increase *p* < 0.001). Treatment with either NAC, MET or the combination of MET+NAC was able to decrease lipid accumulation when compared to the HG control (decrease by 56% and 66% and 64%; *p* < 0.001). MET+NAC treatment was as effective at reducing lipid content as treatment with MET only (Figure 3).

### 3.4. The Combination Effect of MET and NAC to Attenuate ROS Production

**Figure 4:**
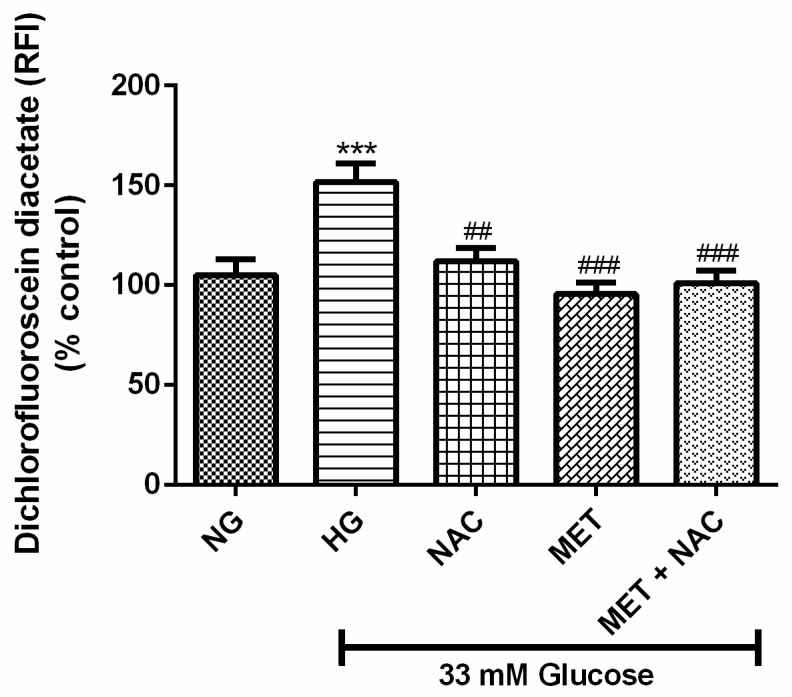
The combination effect of Metformin (MET) and N-acetylcysteine (NAC) on high glucose-induced oxidative stress. 2’, 7’-Dichlorofluorescin diacetate (DCFH-DA) fluorescence as a measurement of reactive oxygen species production was assessed in H9c2 cardiomyoblasts cultured in high glucose (HG; 33 mM) for 24 h. Cells co-treated with NAC (1 mM), metformin (MET) (1 μM) and a combination of MET+NAC attenuated this effect. Cells treated with normal glucose (NG) served as the vehicle control. Data are expressed as the mean ± SEM of 3 independent experiments with each experiment having 3 technical repeats (*n* = 9). Significance is depicted as *** *p* ≤ 0.001 versus NG control, ## *p* ≤ 0.01, ### *p* ≤ 0.001 versus HG control.

High glucose-stimulated oxidative stress is related to accelerated cardiovascular disease. H9c2 cardiomyoblasts, cultured in HG, exhibited a significant increase in intracellular ROS levels in comparison to the NG control (152% vs. 100%; *p* < 0.001). Treatment with either NAC (104%; *p* < 0.01) or MET (95%; *p* < 0.001) or the combination MET+NAC (97%; *p* < 0.001) drastically decreased the production of intracellular ROS when compared to the HG control (Figure 4). The combined use of MET+NAC was as effective as the MET treatment alone.

### 3.5. The Combination Effect of MET and NAC to Diminish Lipid Peroxidation

**Figure 5:**
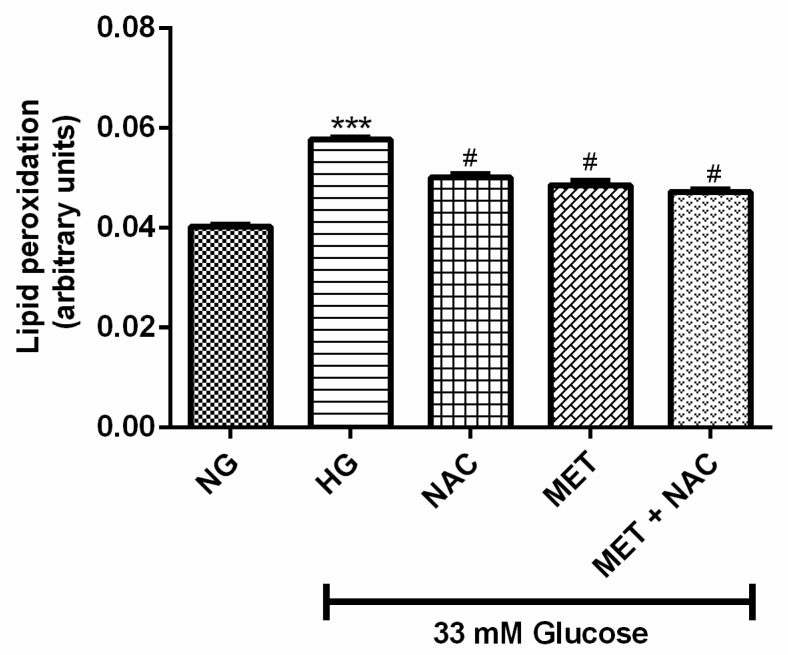
The combination effect of Metformin (MET) and N-acetylcysteine (NAC) on high glucose-induced lipid peroxidation. Malondialdehyde (MDA) formation is a measurement of lipid peroxidation. H9c2 cardiomyoblasts were cultured in high glucose (HG; 33 mM) for 24 h. Cells were then co-treated with NAC (1 mM), metformin (MET) (1 μM) and a combination of MET+NAC. Data are presented as the mean ± SEM of 3 distinct biological experiments with each experiment having 3 technical repeats (*n* = 9). Significance is depicted as *** *p* ≤ 0.001 versus NG control, # *p* < 0.05, versus HG control.

Hyperglycaemia and oxidative stress play a crucial role in the dysregulation of lipid peroxidation (malondialdehyde-MDA) in the diabetic heart. High glucose-induced lipid peroxidation was evident in H9c2 cardiomyoblasts exposed to HG when compared to the NG control (45% increase; *p* < 0.001). The combination of MET+NAC (18% decease; *p* < 0.05) was able to decrease the production of MDA to a similar effect as either NAC (25% decrease; *p* < 0.05) or MET (23% decrease; *p* < 0.05) when compared the HG control (Figure 5). However, the treatments did not reverse lipid peroxidation to the level of the NG control.

### 3.6. The Combination Effect of MET and NAC to Augment SOD Activity And GSH Content

**Figure 6:**
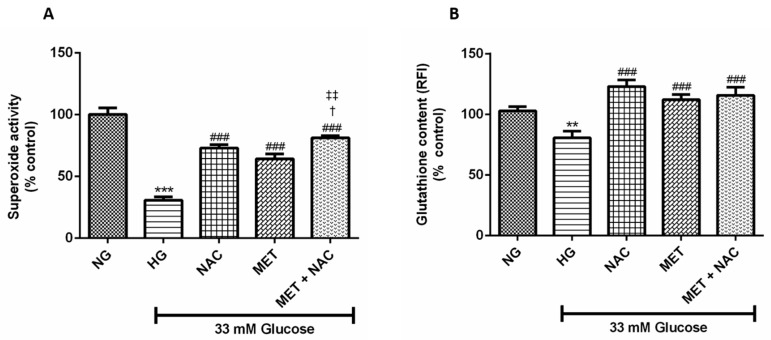
The combination effect of Metformin (MET) and N-acetylcysteine (NAC) on (**A**) superoxide dismutase (SOD) and (**B**) glutathione (GSH) content. H9c2 cardiomyoblasts were cultured in high glucose (HG; 33 mM) for 24 h. Cells were then-treated with NAC (1 mM), metformin (MET) (1 μM) and a combination of MET+NAC. Cells treated with normal glucose (NG) served as the vehicle control. Data are expressed as the mean ± SEM of 3 distinct biological experiments with each experiment having 3 technical replicates (*n* = 9). Significance is depicted as ** *p* ≤ 0.01, *** *p* ≤ 0.001 versus NG control, ### *p* ≤ 0.001 versus HG control, † *p* < 0.05 versus NAC and ‡‡ *p* ≤ 0.01 versus MET.

Increased oxidative stress is the main initiator of cardiac dysfunction in the diabetic heart. This study showed that H9c2 cardiomyoblasts cultured in HG displayed a significant reduction in SOD activity (31% compared to 100% *p* < 0.001) and GSH content (83% compared to 100%; *p* < 0.01) when compared to the normal control. This effect was abolished when cells were treated with NAC (*p* < 0.001), MET (*p* < 0.001) and the combination of MET+NAC (*p* < 0.001); (Figure 6A,B). Treatment with the combination of MET+NAC significantly increased SOD activity when compared to either NAC (*p* < 0.05) or MET (*p* < 0.01) alone.

### 3.7. The Combination Effect of MET and NAC to Modify Mitochondrial Membrane Potential (ΔΨm)

**Figure 7:**
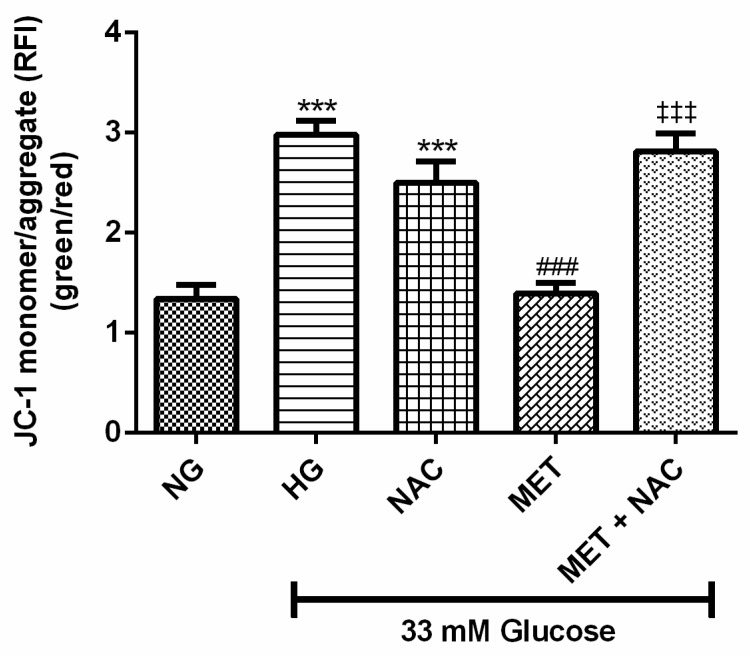
The combination effect of Metformin (MET) and N-acetylcysteine (NAC) on mitochondrial potential. H9c2 cardiomyoblasts were cultured in high glucose (HG; 33 mM) for 24 h. Thereafter, cells were treated with NAC (1 mM), metformin (MET) (1 μM) and a combination of MET+NAC. Cells treated with normal glucose (NG) served as the vehicle control. Data are presented as the mean ± SEM of 3 separate biological experiments with each experiment having 3 technical repeats (*n* = 9). Significance is depicted as *** *p* ≤ 0.001 versus NG control, ### *p* ≤ 0.001 versus HG control and ‡‡‡ *p* ≤ 001 compared to MET.

Impaired mitochondrial potential as a measurement of early apoptosis was assessed in cardiomyoblasts cultured in HG. There was a significant increase in depolarised mitochondria in cells exposed to HG when compared to the NG control (2.8 compared to 1.3, *p* < 0.001). Treatment with MET (1.39; *p* < 0.001) significantly prevented mitochondrial depolarisation, whereas NAC as well as the combination of MET+NAC only showed a trend towards decreased depolarisation (*p* < 0.05, Figure 7).

### 3.8. The Effect of MET and NAC on DNA Fragmentation

**Figure 8:**
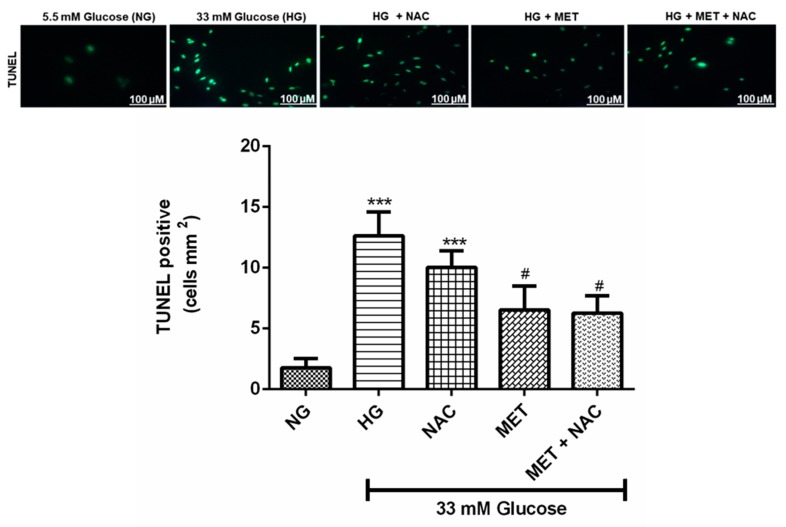
The combination effect of Metformin (MET) and N-acetylcysteine (NAC) on high glucose-induced DNA fragmentation. (A) Photograph microscopy analysis of Tunel positive cells as observed with green fluorescence. (B) Bar graph analysis. H9c2 cardiomyoblasts were cultured in high glucose (HG; 33 mM) for 24 h. Cells were then treated with NAC (1 mM), metformin (MET) (1 μM) and a combination of MET+NAC. Cells treated with normal glucose (NG) served as the vehicle control. Data presented are expressed as the mean ± SEM of 3 separate biological experiments with each experiment having 3 technical repeats (*n* = 9). Significance is depicted as *** *p* ≤ 0.001 versus NG control, # *p* < 0.05.

TUNEL assay was used for the detection of DNA fragmentation, which is a hallmark of apoptosis. In this study, increased TUNEL positive cells were detected in cardiomyoblasts exposed to HG when compared to the NG control (13.63 ± 1.96 compared to 1.75 ± 0.77; *p* < 0.001). Metformin treatment significantly (6.50 ± 1.99; *p* < 0.05) decreased this effect to a similar degree in cells exposed to the combination treatment of MET+NAC (6.25 ± 1.42; *p* < 0.05), whereas NAC treatment showed a trend towards reduced activity when compared to the HG control (10.63 ± 1.96 versus 102.63 ± 1.39). MET+NAC was able to reduce DNA fragmentation to a similar degree as the MET treatment (Figure 8), but none of the treatments reduced DNA fragmentation to the levels of the NG control.

### 3.9. The Effect of MET and NAC on Caspase 3/7 Activity

**Figure 9:**
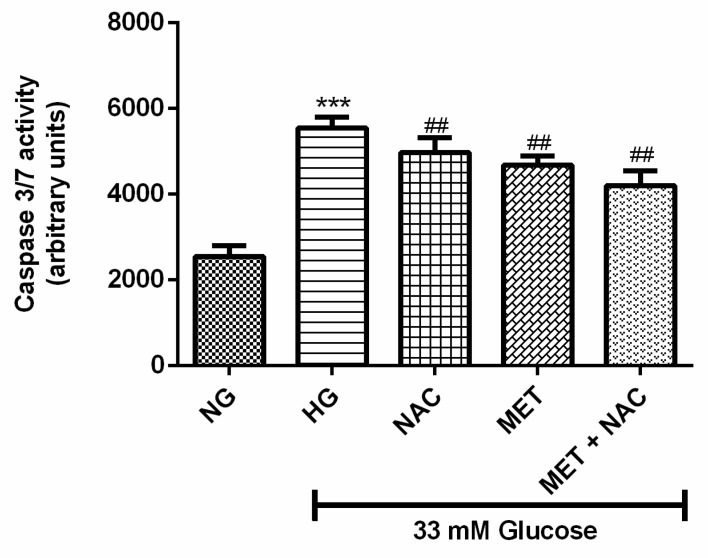
The combination effect of Metformin (MET) and N-acetylcysteine (NAC) on high glucose-induced caspase 3/7 activity. H9c2 cardiomyoblasts were cultured in high glucose (HG; 33 mM) for 24 h. Thereafter, cells were treated with NAC (1 mM), metformin (MET) (1 μM) and a combination of MET+NAC. Cells treated with normal glucose (NG) served as the vehicle control. Results are expressed as the mean ± SEM of 3 independent biological experiments with each experiment having 3 technical replicates (*n* = 9). Significance is depicted as *** *p* ≤ 0.001 versus NG control, ## *p* ≤ 0.01 versus HG control.

Caspase 3/7 activity assay is used to measure cellular apoptotic death. High glucose treatment significantly increased caspase-3/7 activity in H9c2 cardiomyoblasts when compared to the NG control (*p* < 0.001). Treatment with MET+NAC (*p* < 0.01), MET (*p* < 0.01) as well as NAC (*p* < 0.01), was able to decrease caspase activity, however the treatments were unable to completely reduce caspace 3/7 activity to the level of the NG control (Figure 9).

### 3.10. Gene and Protein Expression

#### 3.10.1. The effect of MET and NAC on phosphorylated 5’ AMP-activated protein kinase (AMPK) and nuclear factor-kappa B phosphorylation (p-NF-kB) expression

**Figure 10:**
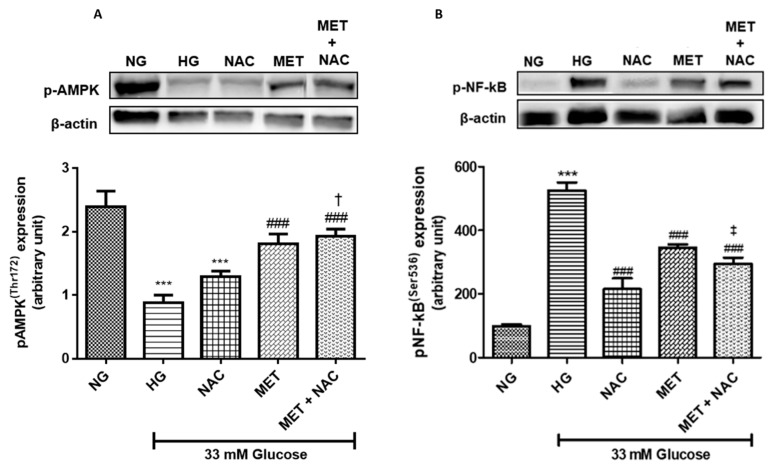
The combination effect of Metformin (MET) and N-acetylcysteine (NAC) on 5’ AMP-activated protein kinase phosphorylation at threonine 172 (p-AMPKThr172) and nuclear factor-kappa B phosphorylation at serine 536 (p-NF-kB (Ser536)). Protein expression of (**A**) p-AMPK(Thr172) and (**B**) p-NF-kB (Ser536) on H9c2 cardiomyoblasts cultured in high glucose (HG; 33 mM) for 24 h. Thereafter, cells were treated with NAC (1 mM), metformin (MET) (1 μM) and a combination of MET+NAC. Cells treated with normal glucose (NG) served as the vehicle control. Results are expressed as the mean ± SEM of 3 independent biological experiments with each experiment having 3 technical replicates (n = 9). Significance is depicted as *** *p* ≤ 0.001 versus NG control, ### *p* ≤ 0.001 versus HG control, † *p* ≤ versus MET and ‡ *p* ≤ 0.05 versus NAC.

The major kinase that plays a role in energy homeostasis is 5’ AMP-activated protein kinase (AMPK), whilst NF-kB, a transcription factor, regulates the expression of genes involved in inflammation in response to increased glucose levels. The expression of p-AMPK(Thr172) in H9c2 cardiomyoblasts was significantly diminished following HG exposure when compared to the NG control (*p* < 0.001). H9c2 cardiomyoblasts exposed to MET (*p* < 0.001) and the combination of MET+NAC (*p* < 0.001) were able to significantly upregulate the expression of p-AMPK(Thr172). The combination treatment was similar to MET treatment only (Figure 10A) but induced significantly higher levels when compared to NAC alone (*p* < 0.05). HG exposed H9c2 cells presented with a significant increase in p-NF-kB(Ser536) expression when compared to cells cultured in NG (*p* < 0.001). NAC (*p* < 0.001), MET (*p* < 0.001), as well as the combination of MET+NAC (*p* < 0.001) were able to significantly attenuate the phosphorylation of NF-kB(Ser536). Interestingly, NAC treatment only was better at attenuating p-NF-kB protein expression compared to either MET or the combination of MET+NAC treatment (*p* < 0.05 against Met+NAC; Figure 10B).

#### 3.10.2. The effect of MET and NAC to increased glucose uptake, through the inhibition of phosphorylated protein kinase C (PKC(Ser660))/insulin receptor substrate-1 (IRS-1(Ser307)) inhibition

**Figure 11:**
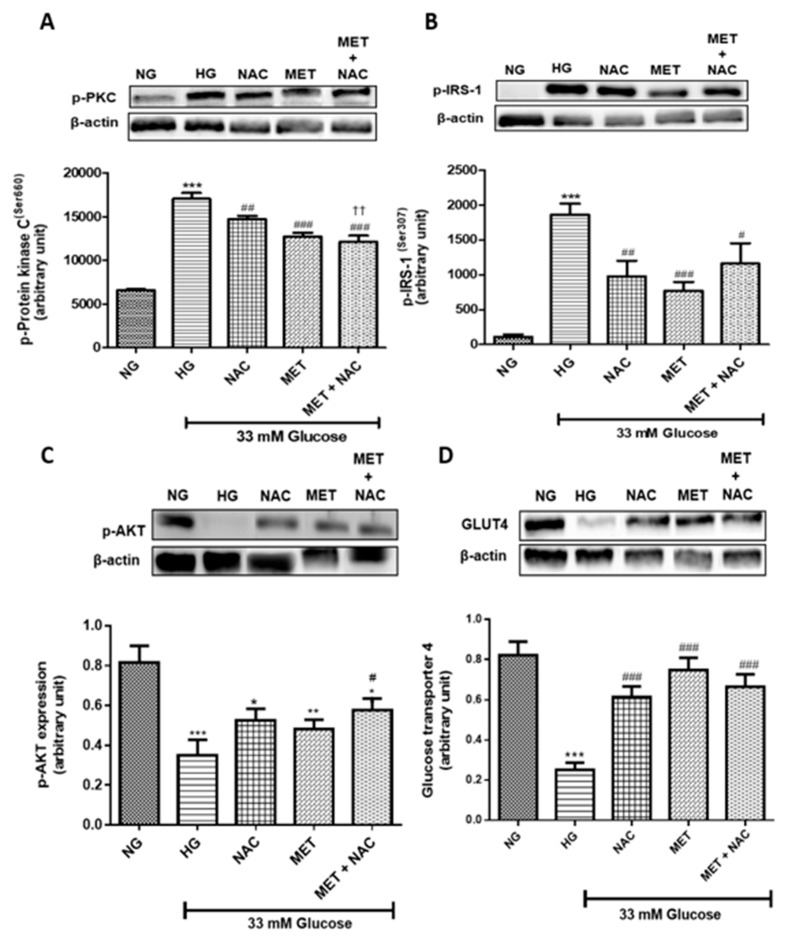
The combination effect of Metformin (MET) and N-acetylcysteine (NAC) on selected targets in the insulin signalling pathway. Protein expression of (**A**) protein kinase C phosphorylation at Serine 660 (p-PKC(Ser660)), (**B**) insulin receptor substrate-1 at Serine 307 (p-IRS-1(Ser307)), (**C**) protein kinase B phosphorylation at Serine 473 (p-AKT(Ser473)) and (**D**) glucose transporter 4 (GLUT4) protein expression of H9c2 cardiomyoblasts cultured in high glucose (HG; 33 mM) for 24 h. Thereafter, cells were treated with NAC (1 mM), metformin (MET) (1 μM) and a combination of MET+NAC. Cells treated with normal glucose (NG) served as the vehicle control. Results are expressed as the mean ± SEM of 3 Independent biological experiments with each experiment having 3 technical replicates (*n* = 9). Significance is depicted as * *p* ≤ 0.05, ** *p* ≤ 0.01, *** *p* ≤ 0.001 versus NG control, # *p* ≤ 0.05, ## *p* ≤ 0.01, ### *p* ≤ 0.01 versus HG control, and ‡‡ *p* ≤ 0.01 versus MET.

Increased serine phosphorylation of protein kinase (p-PKC(Ser660)) has been linked with insulin resistance. H9c2 cardiomyoblasts cultured in HG significantly increased the expression of p-PKC(Ser660) when compared to the NG control (*p* < 0.001). Treatment with NAC (*p* < 0.01), MET (*p* < 0.001) as well as MET+NAC (*p* < 0.001) significantly mitigated the phosphorylation of PKC when compared to the HG control (Figure 11A). Furthermore, serine phosphorylation of insulin receptor substrate-1 (p-IRS-1(Ser307)) inhibits insulin signalling which contributes to peripheral insulin resistance. A significant increase in IRS-1(Ser307) phosphorylation was observed in H9c2 cardiomyoblasts exposed to HG when compared to the NG control (*p* < 0.001). Treatment with NAC (*p* < 0.01), MET (*p* < 0.001) and the combination of MET+NAC (*p* < 0.05) were able to alleviate this effect (Figure 11B).

Phosphorylated protein kinase B (pAKT) signalling plays a central role in insulin-stimulated GLUT4 expression. In the present study, H9c2 cardiomyoblasts cultured in HG presented with a significant decrease in the expression of AKT when compared to the NG control (*p* < 0.001). Interestingly, MET, NAC showed an increasing trend, whereas the combination (MET+NAC) treatment significantly (*p* < 0.05) increased AKT expression (Figure 11C). Similarly, this study showed that GLUT4 expression, which is a major transporter of glucose in the heart, was affected by HG exposure. HG resulted in a significant decrease in GLUT4 expression when compared to cells cultured in NG (*p* < 0.001). However, this reduction was significantly reversed following treatment with NAC (*p* < 0.001), MET (*p* < 0.001) and that of MET+NAC (*p* < 0.001) when compared to the HG control (Figure 11D).

## 4. Discussion

Due to an ageing population and amplified diabesity epidemic, HF is increasing worldwide [24]. Current consensus is that chronic hyperglycaemia and hyperlipidaemia are the main initiators of augmented oxidative stress. This imbalance between the production of free radicals and the inability of the body’s endogenous antioxidant system to counteract or detoxify reactive products is known to increase the risk of premature HF [25,26,27].

To date, scientific evidence has shown that current glucose-lowering drugs, such as MET, are able to protect the myocardium against augmented oxidative stress [28,29,30,31]. However, the ability of MET to protect the myocardium may be reduced over time, emphasising the need to incorporate alternative therapies as an adjunct to improve the endogenous antioxidant status of the diabetic heart. As such, studies have presented data reporting on the preventive properties of NAC in hyperglycaemia-induced oxidative stress [11,32,33,34,35]. Dludla (2018) performed a systematic review confirming the ameliorative properties of NAC and highlighted that there is a scarcity of data reporting on the comparative effect of NAC with common glucose-lowering therapies on the diabetic myocardium [11]. Therefore, this study set out to investigate if the combined use of MET and NAC is better than the use of MET alone to protect the diabetic myocardium against aberrant hyperglycaemia-induced oxidative stress and cell apoptosis induced by the hyperglycaemic state. 

In a systematic review performed by Kajbaf and colleagues (2016), the authors reported that MET and NAC therapy are beneficial within a disease state [29]. The authors further elaborated on the plasma concentrations of MET and NAC, further stating that for MET and NAC to have therapeutic effects, MET plasma concentrations must be in the range of 0.129-90 mg/L, while that of NAC equal 0.02 mM. The latter can be used to guide in vivo dose experiments. Unfortunately, there is no practical guidelines for dose translation experiments from human to in vitro dose and as such, the selected doses for NAC and MET were based on previous findings from our group [7,19,20]. Therefore, in this study H9c2 cells were exposed to 1 µM MET, a clinically relevant dose that closely mimics metformin serum levels, while NAC was used at 1 mM, an accomplished in vitro experimental dose. In this context, H9c2 cells exposed to HG were either treated alone or in combination with MET and NAC, as previously reported [7]. Consequently, experimental exposure of cardiomyocytes to HG increased oxidative stress as it could be observed with a 1.5-fold increased DCFH-DA level, while SOD and GSH levels were significantly decreased, suggesting a depletion of intracellular antioxidative mechanisms. This study presented data on NAC, MET and the combination of MET+NAC’s ability to reverse accelerated oxidative stress which is known to induce tissue damage. Findings from this study further support published data confirming that administration of NAC replenishes GSH and SOD levels, while reducing oxidative stress markers in the myocardium of infarcted rat hearts [35,36,37]. N-acetylcysteine is a precursor to the amino acid L-cysteine and consequently the antioxidant GSH. Therefore, we were expecting to detect an enhanced effect with the amalgamation of MET and NAC, however no significant additive or synergistic effects were observed. In saying that, Dludla (2018) reported that the effect of NAC to display health promoting properties could possibly be dependent on the dose selection [11] and therefore, we speculate that the correct combination of mixing the doses of MET and NAC might possibly be better at replenishing intracellular GSH. On the other hand, the results suggested that markers of late stage tissue damage, such as the end products of lipid peroxidation (TBARS) and late stage apoptosis as indicated by caspase 3/7 activation and DNA damage (Tunel assay), were only partially reversed by the treatments. This damage was likely incurred during the initial HG treatment and it could be speculated that MET and NAC treatment during this initial period of HG exposure may be able to provide more comprehensive protection of these cells.

Apart from abnormal redox cycling induced by chronic glucose exposure, excessive amounts of ROS will interfere with cellular processes and oxidatively degrade membrane lipids in a process called lipid peroxidation. As such, lipid peroxidation can be seen as one of the major mechanisms associated with accelerated myocardial damage in the diabetic state. In a study published in 2013 by Basha and Priscilla, the authors showed that NAC pre-treatment at a dose of 10 mg/kg daily for 14 days in rats displayed robust antioxidant properties against isoproterenol induced myocardial lipid peroxidation and mitochondrial dysfunction similarly, while modulating calcium handling during ischemic reperfusion injury [38]. In another study, Nagoor and Meeran (2011) found that treatment of rats with 10 mg/kg, instead of 5 mg/kg, of NAC was more effective in reducing lipid peroxidation in isoproterenol-induced myocardial infarcted rats [33]. In the current study we showed that NAC treatment was able to ameliorate lipid peroxidation with a similar effect to that of the combined treatment of MET and NAC. Interestingly, the observed effect, though statistically significant, was once again not additive or synergistic and we speculated that a higher dose of NAC might have produced a more marked effect.

Apart from increased oxidative stress and lipid peroxidation, intracellular lipid accumulation plays an important role in the development of cardiac dysfunction [8]. The heart uses larger amounts of fatty acids as an energy source (70%) in order to maintain the physiological balance between lipid uptake and oxidation [8]. During the diabetic state, these carefully controlled processes are altered, resulting in the accumulation of lipid, while using exclusively fatty acid oxidation as an energy source [7]. This will then lead to a chronic altered state with decreased ATP production and energy depletion of the failing heart. In our previous study, we confirmed such a phenomenon, showing how increased fatty acid utilisation after chronic glucose exposure decreased metabolic activity [7]. Consistent with previously reported data on myocardial energy efficiency, our results showed that treatment of H9c2 cells with HG induced a significant decrease in metabolic content and GU, while increasing lipid accumulation. It is acknowledged that MET inhibits complex 1, thereby preventing mitochondrial ATP production. However, this effect can be reversed by a key kinase, AMPK, a master energy regulator that is also known to modulate lipid metabolism. In a disease state, during reduced ATP levels, AMPK is phosphorylated at Thr172, which results in the activation of AMPK and in turn inhibits anabolic processes while catabolic processes and ATP production is increased [39]. The latter finding was supported by Hu (2016) and in the current study, we showed that MET enhanced phosphorylation levels of AMPK in H9c2 cells which resulted in the alteration of cellular redox status and the reversal of HG-induced inhibition of ATP content [40]. Furthermore, Hu (2016) continued and showed that MET was able to activate AMPK in H9c2 cells at 1 mM, 5 mM and 10 mM. However, in this study we showed that MET at a concentration as low as 1 µM was able to increase phosphorylation of AMPK which correlated to an increased in ATP production in cultured cardiomyocytes. This study was also the first to show that the combinatory use of MET plus NAC was significantly more effective than the use of NAC alone, and showed a trend towards increasing energy efficiency and GU compared to MET alone. This effect of the combination drugs was confirmed by the reduction of lipid accumulation, which was similar to that of MET treatment.

It is further acknowledged that glucose homeostasis is maintained by insulin which facilitates the uptake of glucose into peripheral tissue via the GLUT4 transporter. Improved glucose disposal accounts for the redistribution of GLUT4 to the plasma membrane and subsequent regulation and maintenance of glucose homeostasis. Mechanistically, it is known that the NF-kB(Ser536)/p-PKC(Ser660)/p-IRS(Ser307) signalling pathway plays a major role in regulating glucose homeostasis in cardiac cells, while p-AMPK/p-AKT signalling ensures cellular homeostasis through increased GLUT4 uptake (41). As such, increased p-PKC(Ser660)/p-IRS(Ser307) signalling has been linked to augmented lipid accumulation and decreased GU in the diabetic heart [41]. Furthermore, activated p-AMPK and p-AKT are key mediators in salvaging glucose-induced cardiac injury in adult cardiomyocytes [41]. Our findings showed that the combination of MET and NAC was able to enhance GU by reversing the inhibitory effect that p-NF-kB(Ser536)/p-PKC(Ser660)/p-IRS(Ser307) signalling has on p-AMPK(Thr172)/p-AKT(Ser473) ability to increase GU. Interestingly, we found that NAC was able to mediate the observed effect through activation and phosphorylation of p-AKT. Next, we aimed to investigate the effect of MET and NAC on preventing cellular apoptosis. Results from this study were the first to show that the combination of MET and NAC showed improved activity in preventing DNA breaks. NAC treatment had a comparable effect in preventing cardiac apoptosis as compared to the use of MET alone, once again confirming NACs ability to decrease hyperglycaemia-induced cellular apoptosis.

## 5. Conclusions

The findings from this study offer plausible validation that NAC was able to attenuate high glucose-induced oxidative stress, lipid peroxidation and subsequent myocardial apoptosis in H9c2 cardiomyoblasts. However, though effective, the combination of MET and NAC did not diminish the protective effects of the MET treatment alone, which makes the combination of MET and NAC a promising candidate to further investigate their potential to protect the diabetic heart from developing cardiomyopathy. In addition, based on the results obtained we speculate that the combinatory effect of NAC and MET might be more beneficial in improving glucose uptake and increasing the endogenous antioxidant activity. If this holds true then such combination therapy would be worth pursuing, however, this requires further investigation. The authors acknowledge the following limitations to the study; (1) that the efficacy of the combined treatment was only tested in a single H9c2 cardiomyoblast model and (2) lipid toxicity is a phenomenon that occurs over a period of time and that the present study only investigated a single episode of hyperglycaemia. As such, future studies would be needed to confirm such findings on an in vivo rat model.
